# Correction: Aggarwal, M. 2,2-Diphenethyl Isothiocyanate Enhances Topoisomerase Inhibitor-Induced Cell Death and Suppresses Multi-Drug Resistance 1 in Breast Cancer Cells. *Cancers* 2023, *15*, 928

**DOI:** 10.3390/cancers18172850

**Published:** 2026-09-03

**Authors:** Monika Aggarwal

**Affiliations:** Department of Oncology, Lombardi Comprehensive Cancer Center, Georgetown University, Washington, DC 20007, USA; monilaagg@gmail.com

## Error in Figure

In the original publication [1], there was a mistake in Figure 5C as published. The wrong image was used for the mutant p53 control (GAPDH) for the AU565 cell line. The corrected Figure 5C appears below. The authors state that the scientific conclusions are unaffected. This correction was approved by the Academic Editor. The original publication has also been updated.

## Figures and Tables

**Figure 5:**
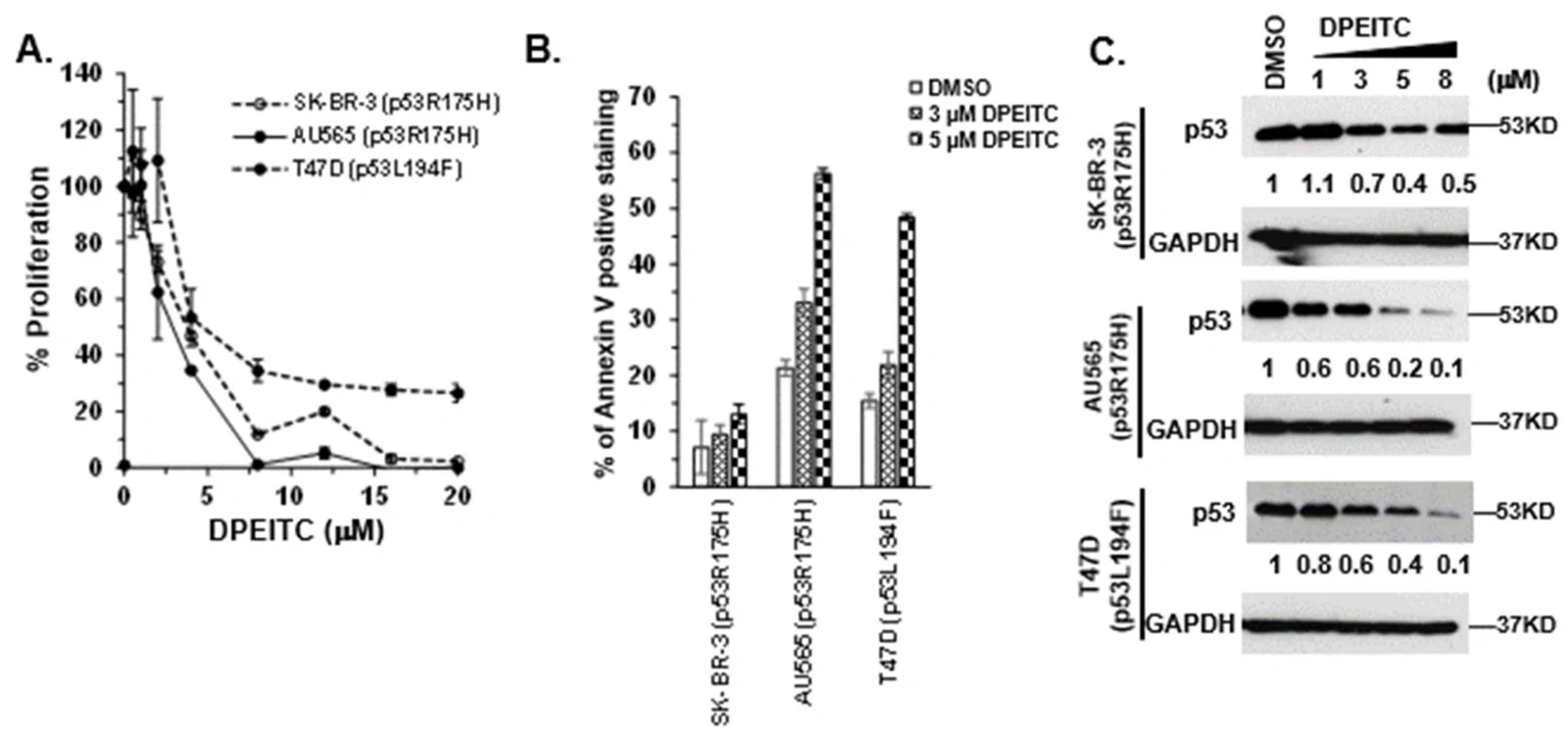
DPEITC inhibits cell proliferation, induces apoptosis, and affects p53 mutant expression levels in HER2_+_ and Luminal A breast cancer cell lines. HER2+ SK-BR-3 (p53^R175H^), AU565 (p53^R175H^), and Luminal A T47D (p53^L194F^) cell lines were treated with DMSO or DPEITC for 72 h and evaluated for percent cell proliferation (**A**) or apoptosis (**B**). (**C**) Cell lysates of HER2+ and Luminal A breast cancer cell lines treated with DMSO or the indicated concentration of DPEITC for 3 h were analyzed by Western blotting. Experiments were performed in triplicate. Error bars represent SD.
