# Supplementary material for: 2,2-Diphenethyl Isothiocyanate Enhances Topoisomerase Inhibitor-Induced Cell Death and Suppresses Multi-Drug Resistance 1 in Breast Cancer Cells
Source: Cancers (Basel). 2023 Feb 1;15(3):928. doi: 10.3390/cancers15030928 (PMC9913484; doi:10.3390/cancers15030928)
Supplement: Supplementary file 1 [file cancers-15-00928-s001.zip › cancers-2188398-File S1 Original Blots.pdf]

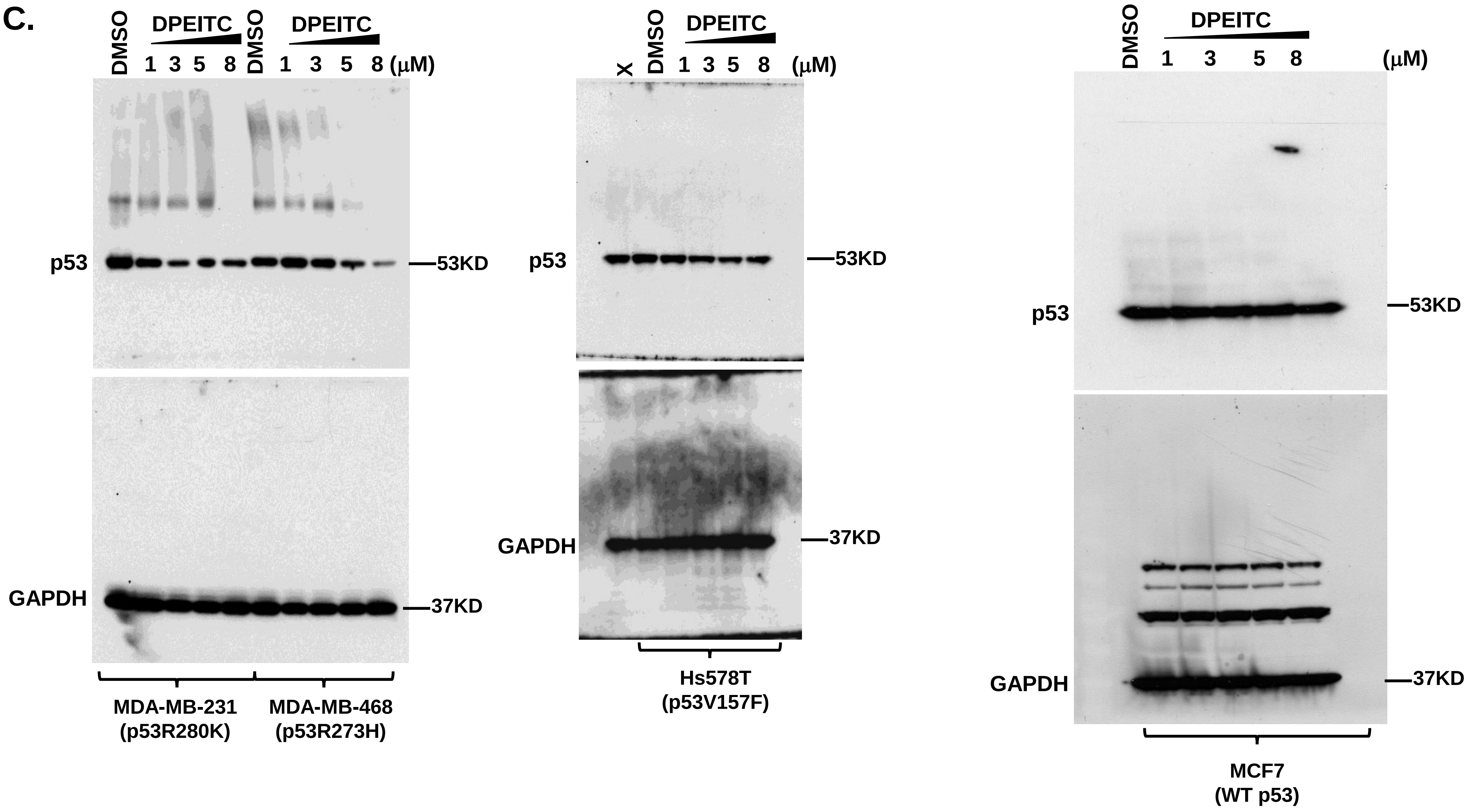
C.
DMSO
DPEITC
1 3 5 8
DMSO
DPEITC
1 3 5 8 (μM)
p53
53KD
GAPDH
37KD
MDA-MB-231
(p53R280K)
MDA-MB-468
(p53R273H)
X
DMSO
DPEITC
1 3 5 8 (μM)
p53
53KD
GAPDH
37KD
Hs578T
(p53V157F)
DMSO
DPEITC
1 3 5 8
(μM)
p53
53KD
GAPDH
37KD
MCF7
(WT p53)

Figure 1

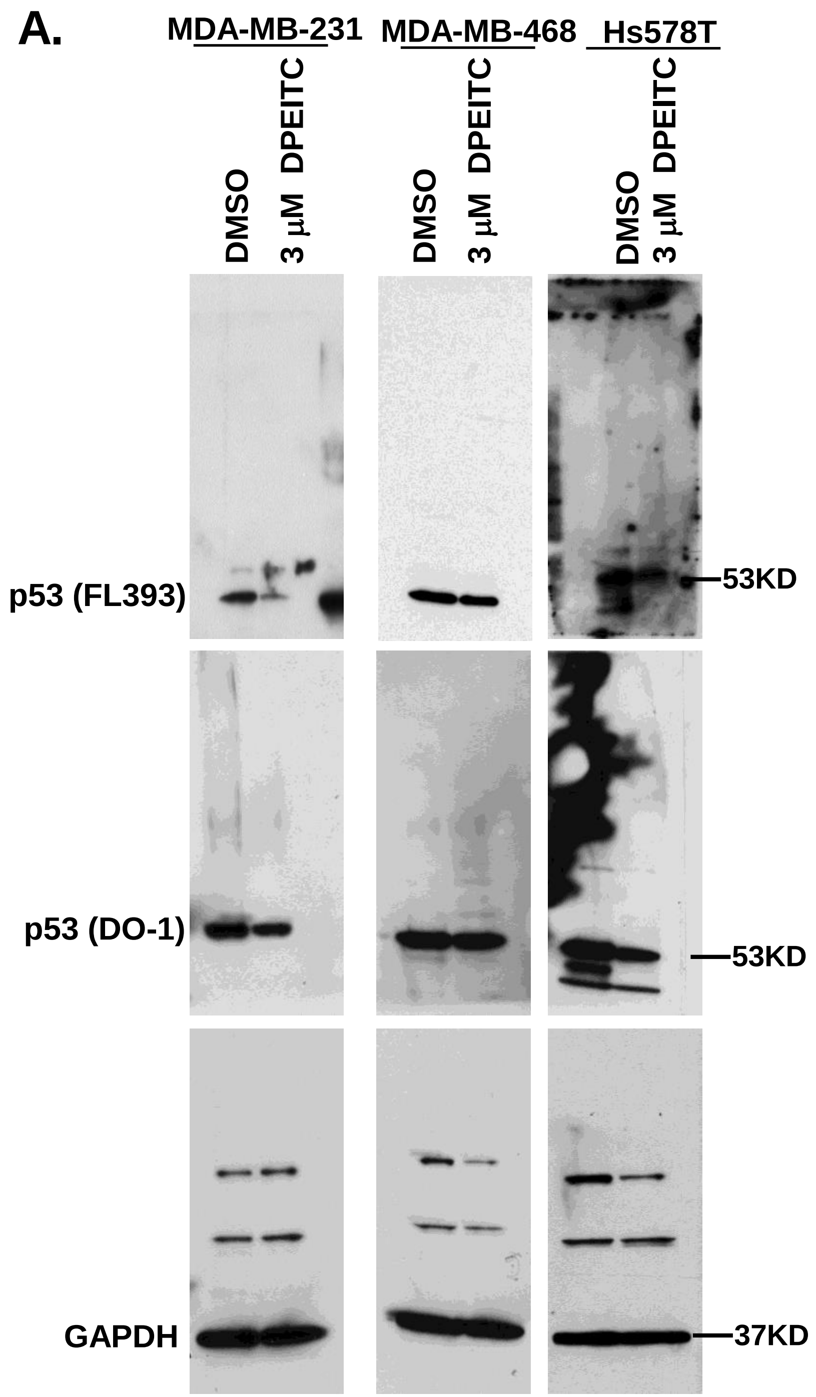
A.
MDA-MB-231
MDA-MB-468
Hs578T
DMSO
3 μM DPEITC
DMSO
3 μM DPEITC
DMSO
3 μM DPEITC
p53 (FL393)
p53 (DO-1)
GAPDH
53KD
53KD
37KD

Figure 2

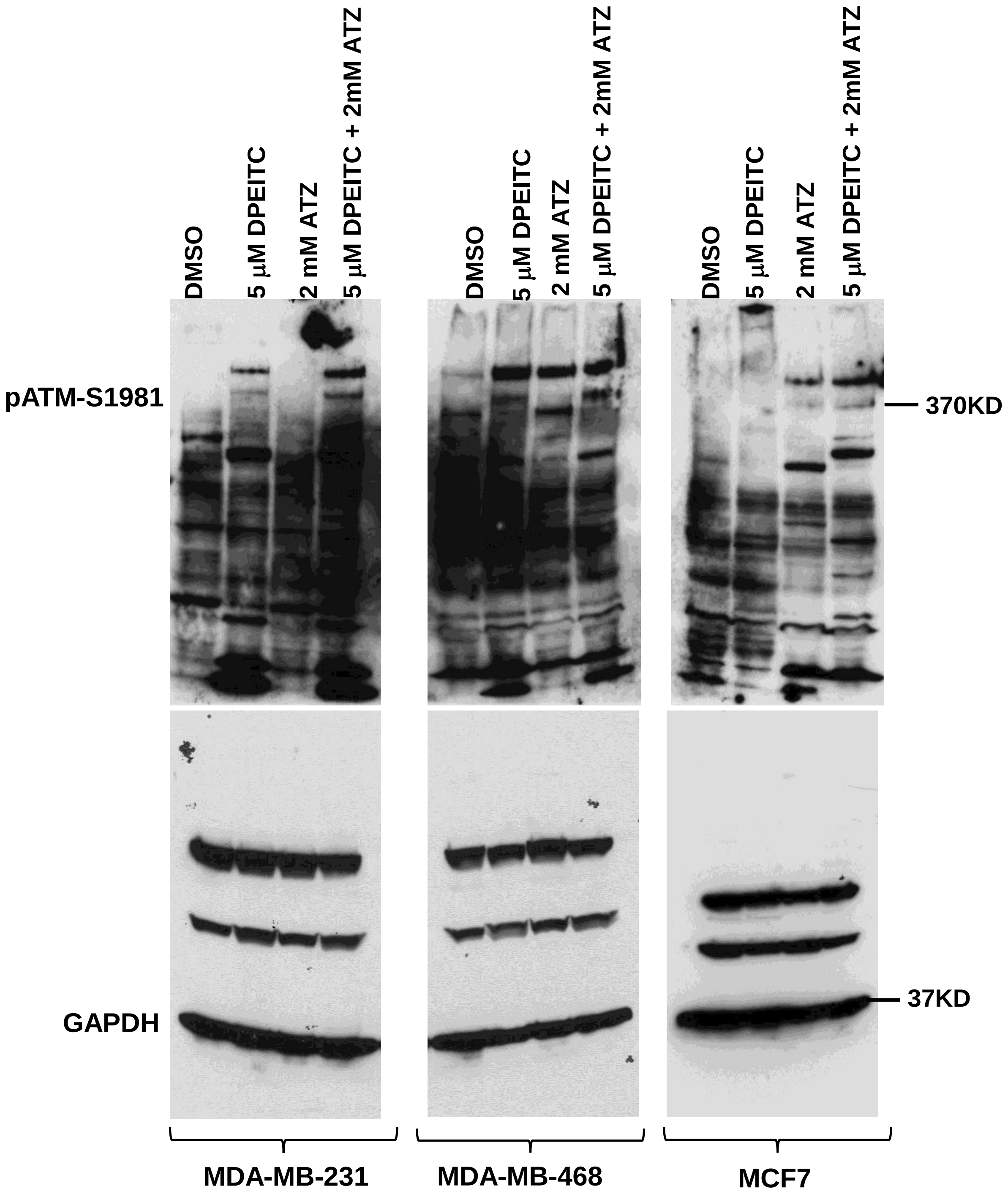
DMSO
5 µM DPEITC
2 mM ATZ
5 µM DPEITC + 2mM ATZ
pATM-S1981
370KD
GAPDH
37KD
MDA-MB-231
MDA-MB-468
MCF7

Figure 4

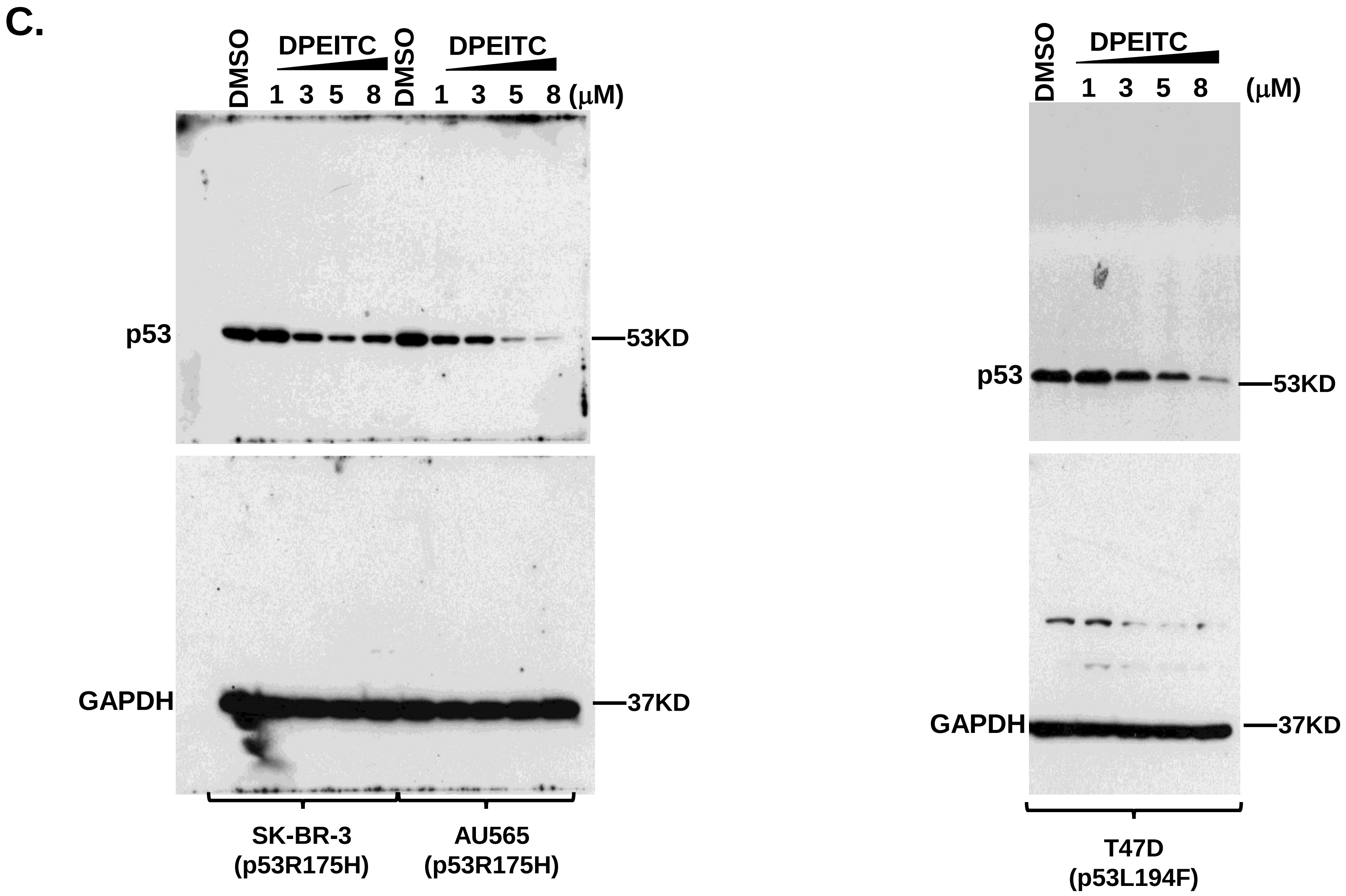
C.
DMSO
DPEITC
1 3 5 8
DMSO
DPEITC
1 3 5 8 (μM)
p53
53KD
GAPDH
37KD
SK-BR-3
(p53R175H)
AU565
(p53R175H)
DMSO
DPEITC
1 3 5 8
(μM)
p53
53KD
GAPDH
37KD
T47D
(p53L194F)

Figure 5

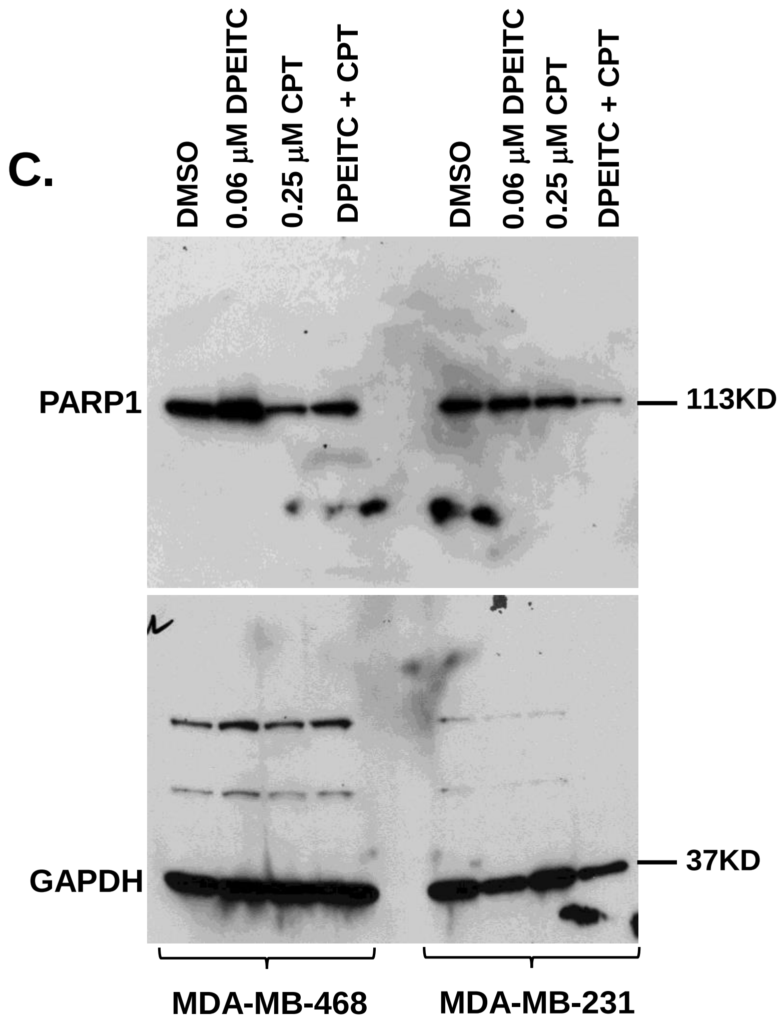
C.
DMSO
0.06 μM DPEITC
0.25 μM CPT
DPEITC + CPT
DMSO
0.06 μM DPEITC
0.25 μM CPT
DPEITC + CPT
PARP1
113KD
GAPDH
37KD
MDA-MB-468
MDA-MB-231

Figure 7

B.

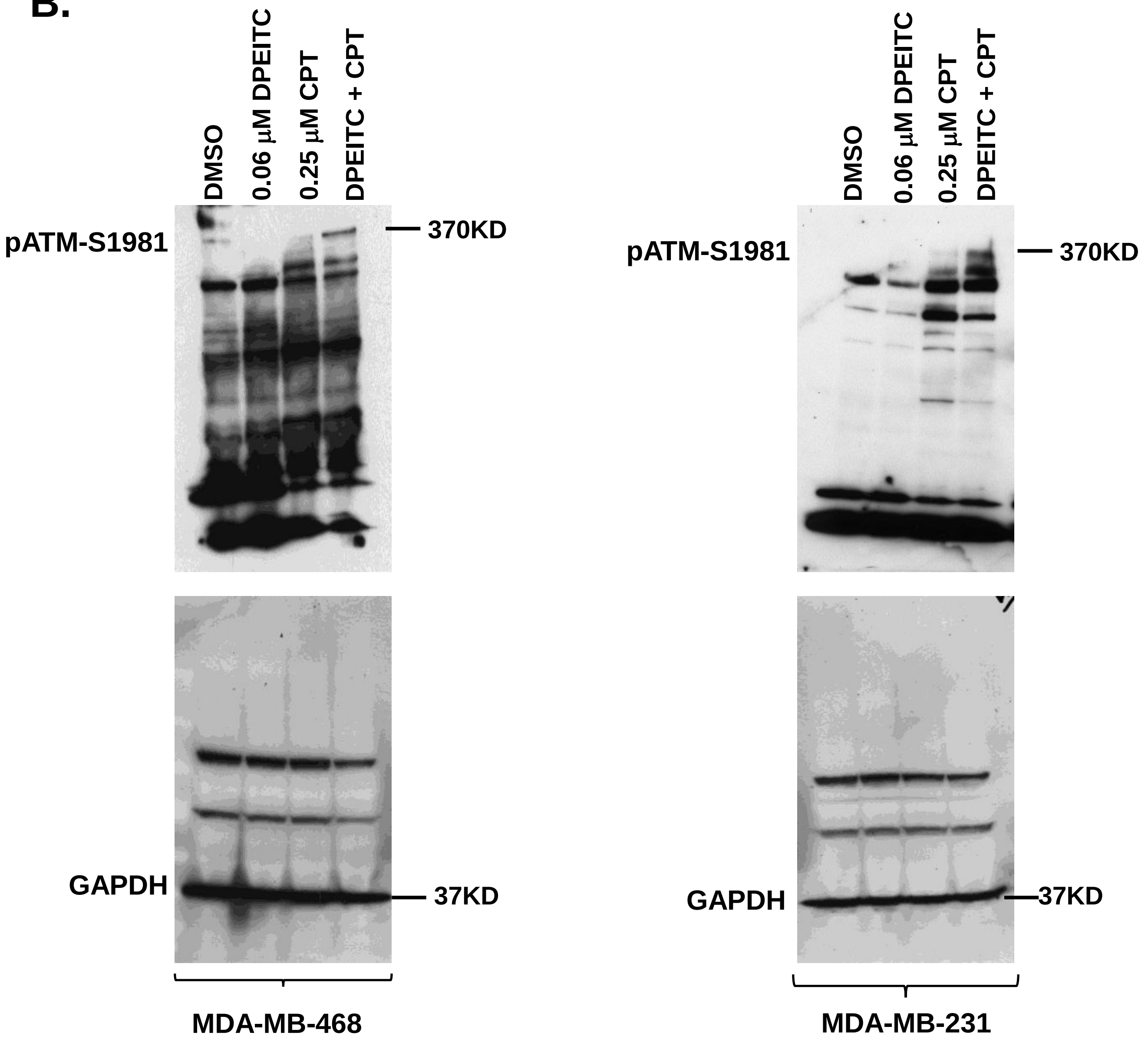
DMSO
0.06 µM DPEITC
0.25 µM CPT
DPEITC + CPT
pATM-S1981
370KD
GAPDH
37KD
MDA-MB-468
DMSO
0.06 µM DPEITC
0.25 µM CPT
DPEITC + CPT
pATM-S1981
370KD
GAPDH
37KD
MDA-MB-231

Figure 8

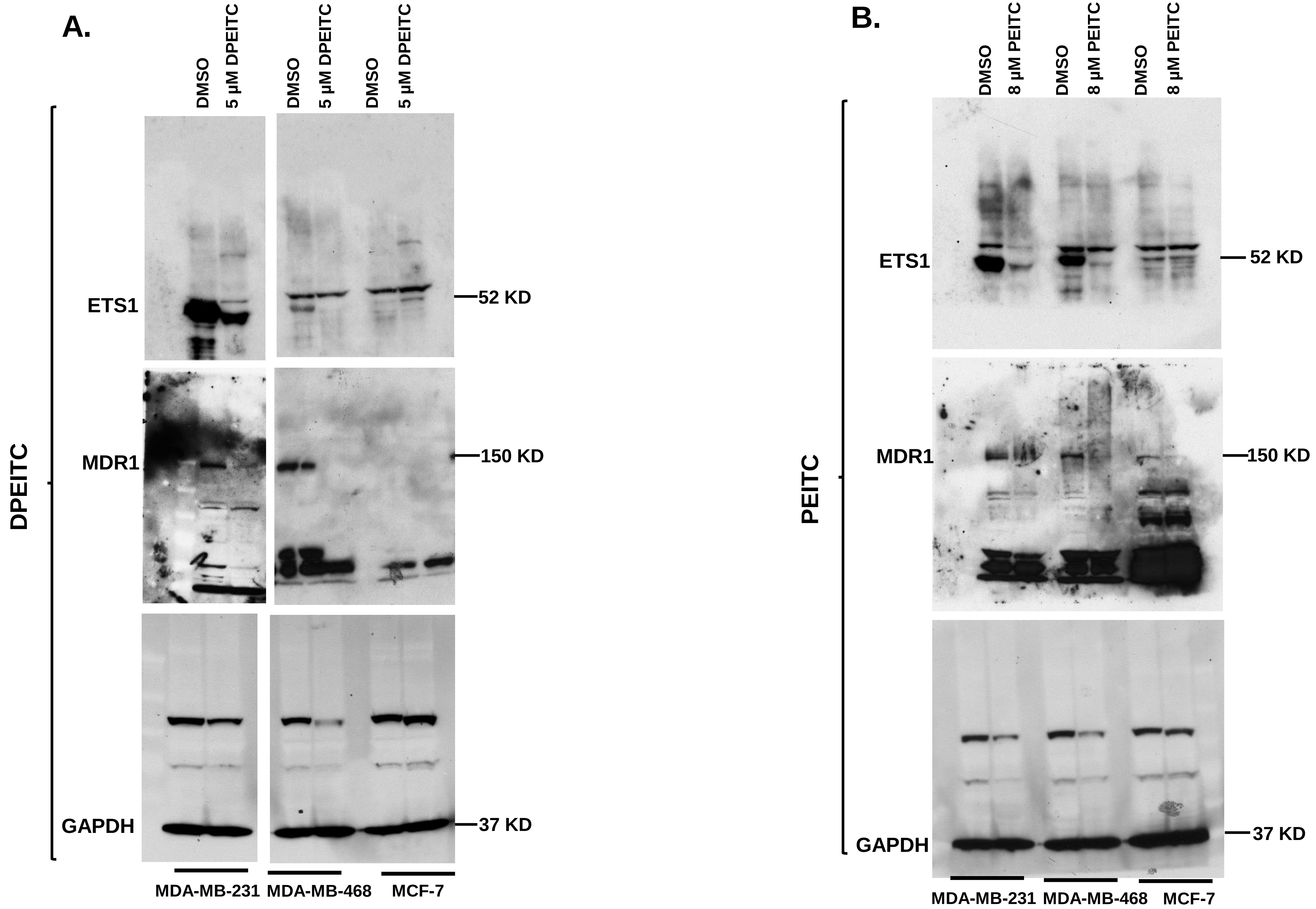
A.
DMSO
5 µM DPEITC
DMSO
5 µM DPEITC
DMSO
5 µM DPEITC
DPEITC
ETS1
52 KD
MDR1
150 KD
GAPDH
37 KD
MDA-MB-231
MDA-MB-468
MCF-7
B.
DMSO
8 µM PEITC
DMSO
8 µM PEITC
DMSO
8 µM PEITC
PEITC
ETS1
52 KD
MDR1
150 KD
GAPDH
37 KD
MDA-MB-231
MDA-MB-468
MCF-7

Figure 9

A.

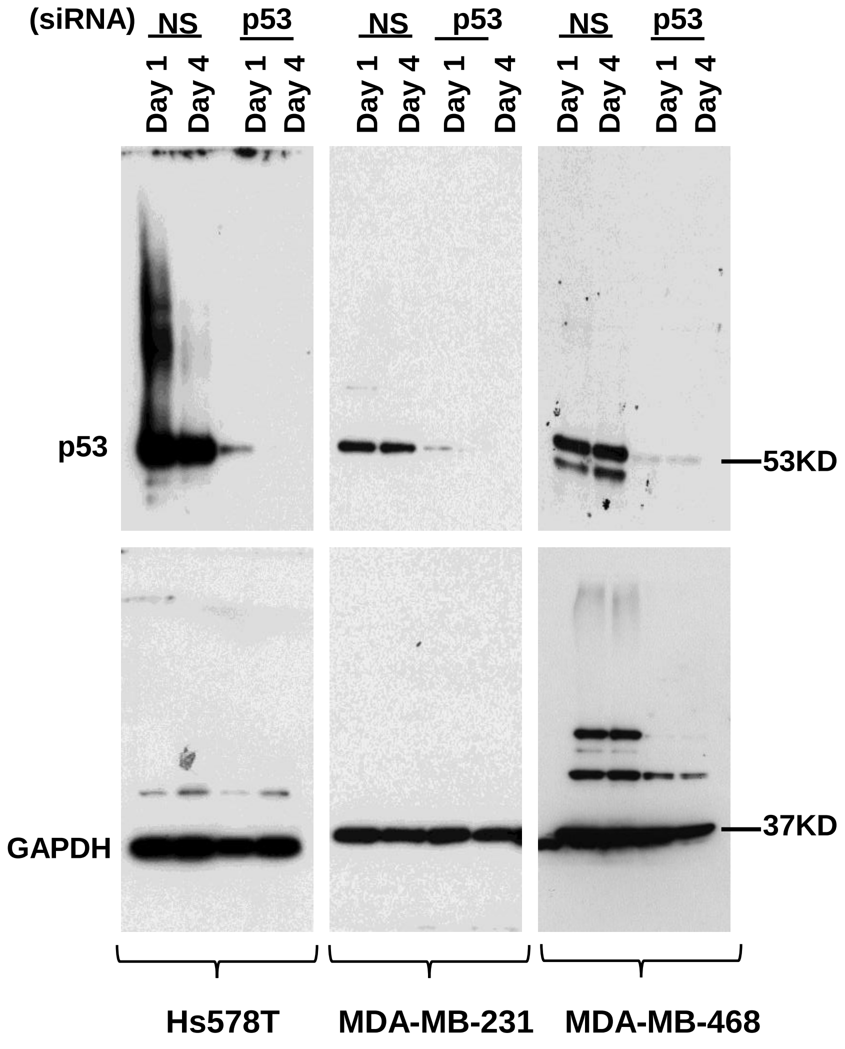

Supplementary Figure S3

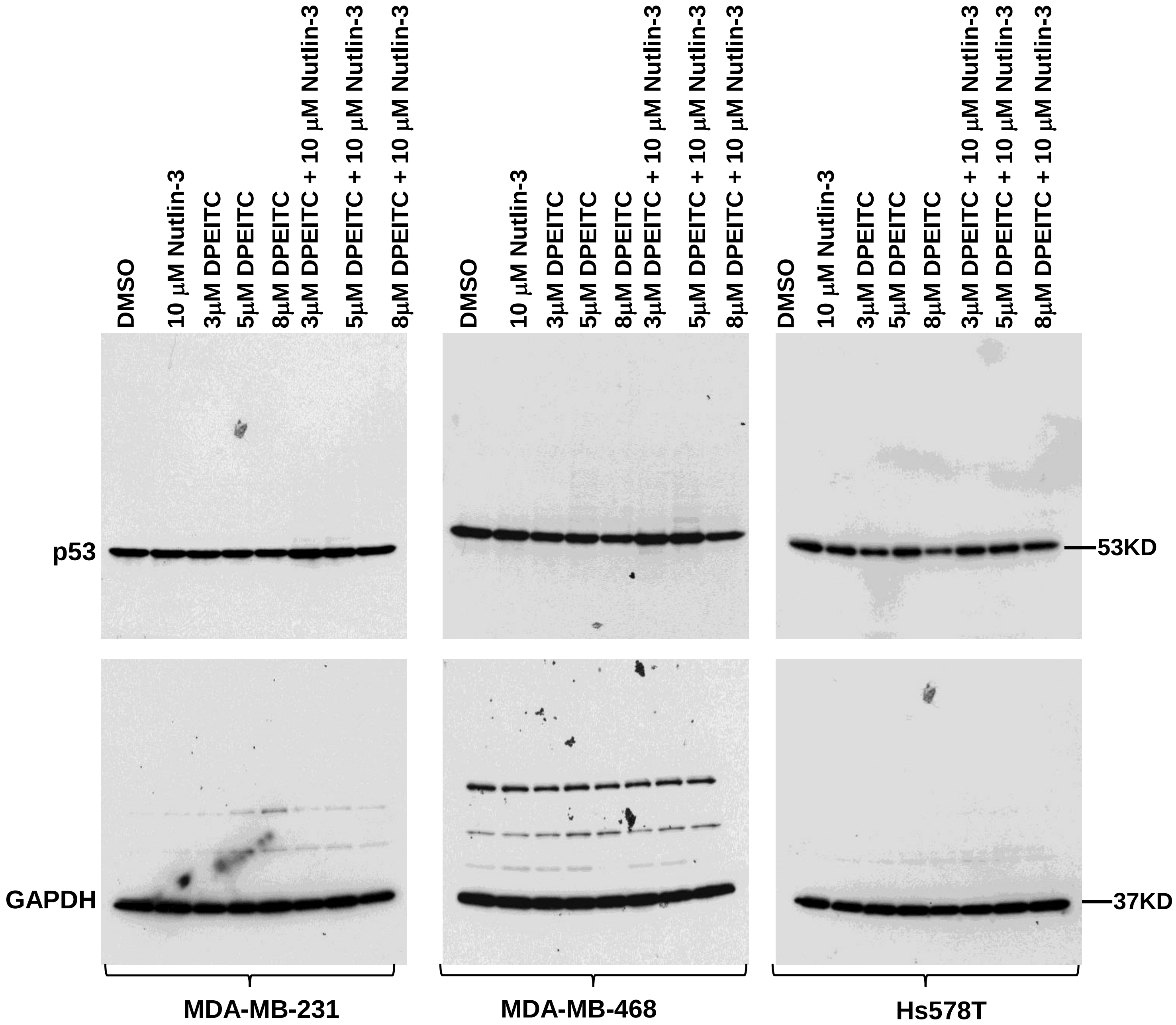

Supplementary Figure S4

C.

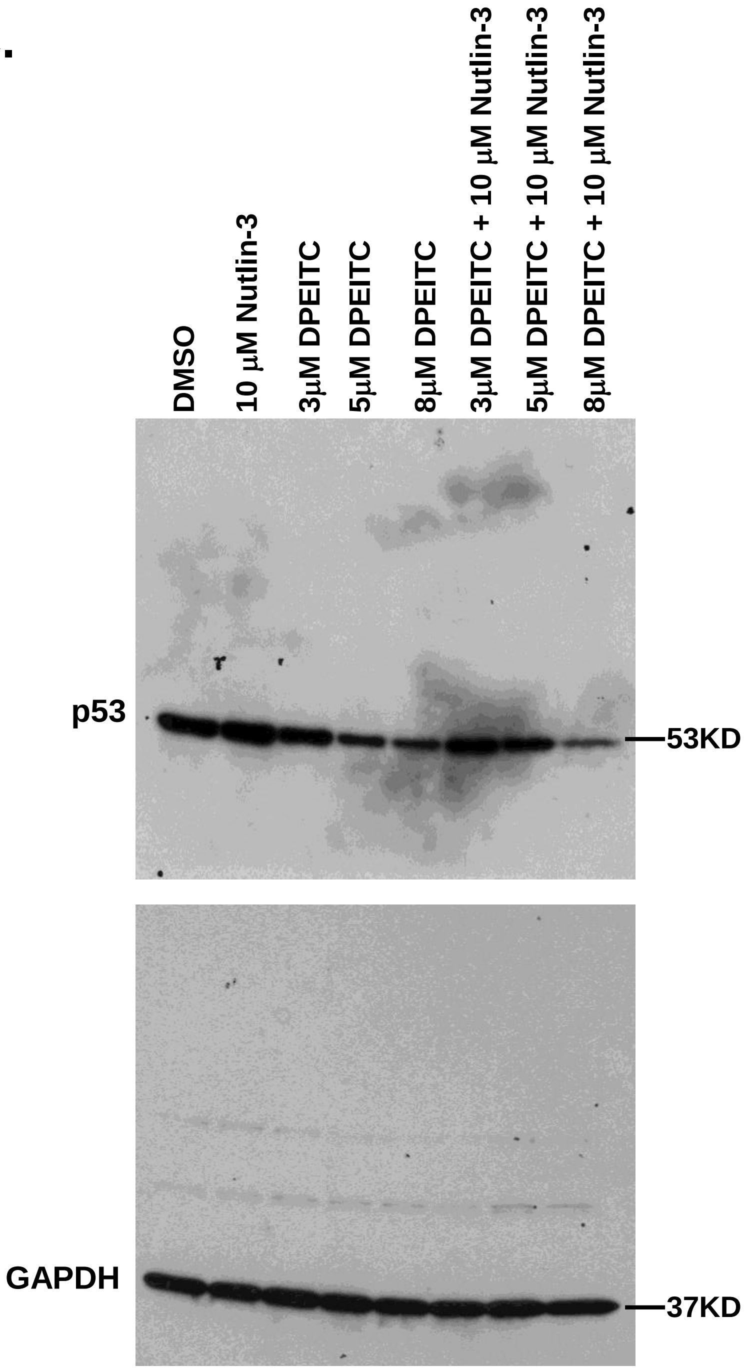

Supplementary Figure S7
