# Supplementary material for: 2,2-Diphenethyl Isothiocyanate Enhances Topoisomerase Inhibitor-Induced Cell Death and Suppresses Multi-Drug Resistance 1 in Breast Cancer Cells
Source: Cancers (Basel). 2023 Feb 1;15(3):928. doi: 10.3390/cancers15030928 (PMC9913484; doi:10.3390/cancers15030928)
Supplement: Supplementary file 1 [file cancers-15-00928-s001.zip › cancers-2188398-supplementary.pdf]

cancers

MDPI

Supplementary Materials

2,2-Diphenethyl Isothiocyanate Enhances Topoisomerase Inhibitor-Induced Cell Death and Suppresses Multi-Drug Resistance 1 in Breast Cancer Cells

Monika Aggarwal

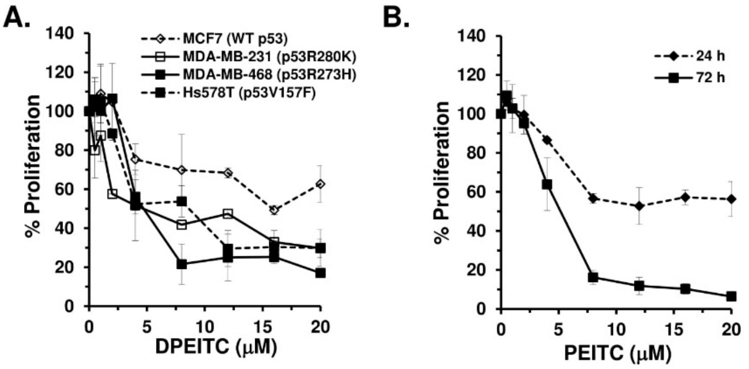

Figure S1. Effects of DPEITC on the proliferation of TNBC cell lines expressing WT p53 or mutant p53. (**A**) MCF7 (WT p53), MDA-MB-231 ($p53^{R280K}$), MDA-MB-468 ($p53^{R273H}$), and Hs578T ($p53^{V157F}$) cells were treated with DMSO or DPEITC for 24 h. Percent cell proliferation was determined by the WST-1 assay. (**B**) Hs578T ($p53^{V157F}$) cells were treated with DMSO or PEITC for 24 h or 72 h. Percent cell proliferation was determined by the WST-1 assay. Experiment were performed in triplicate. Error bars represents SD.

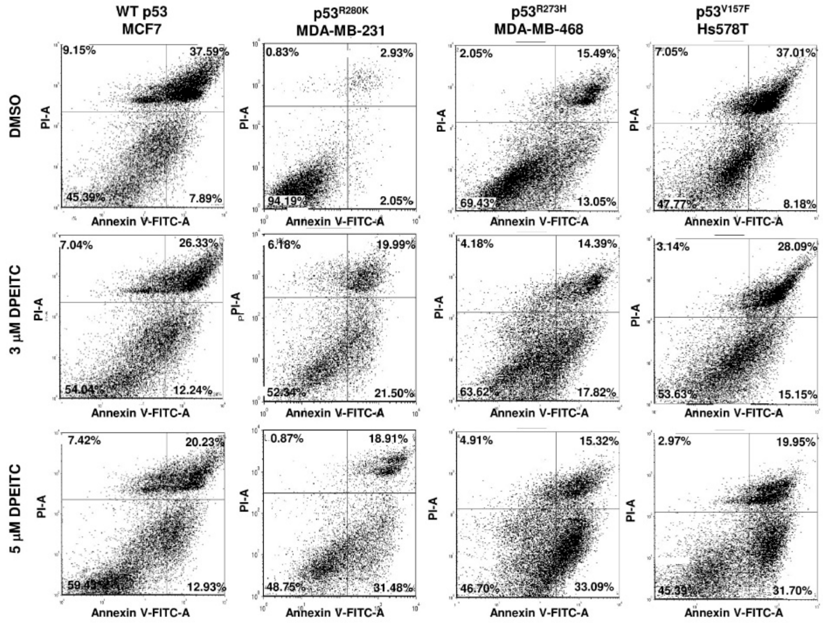

Figure S2. DPEITC induces apoptosis in TNBC cell lines with mutated p53. MCF7 (WT p53), MDA-MB-231 ($p53^{R280K}$), MDA-MB-468 ($p53^{R273H}$), and Hs578T ($p53^{V157F}$) cells were treated with DMSO or DPEITC for 72 h and analyzed for apoptosis. Representative flow cytometry images are shown.

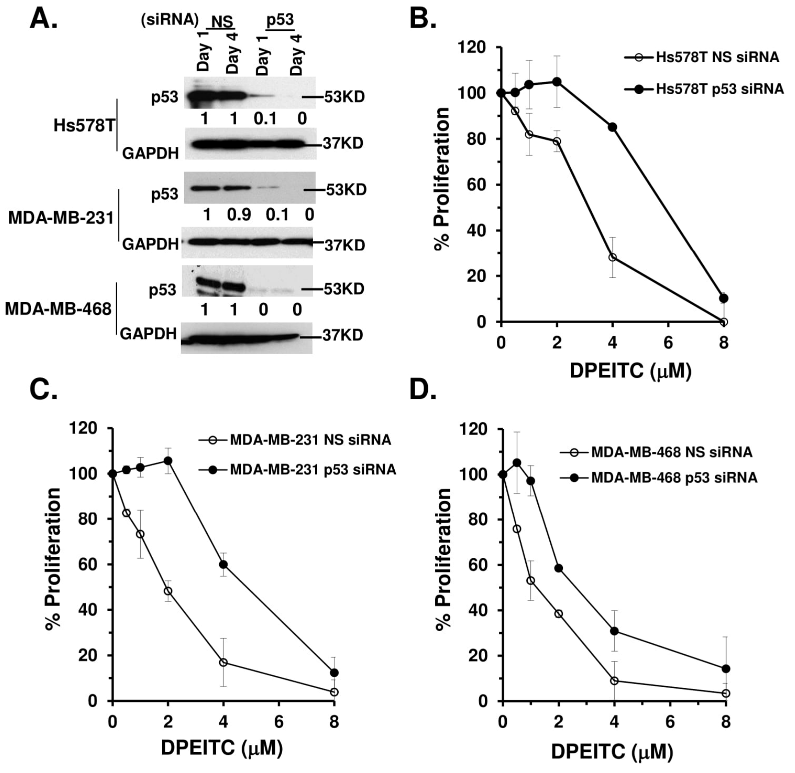

Figure S3. DPEITC inhibits cell proliferation in a mutant p53-specific manner in TNBC cell lines. **(A)** Cell lysates of MDA-MB-231 ($p53^{R280K}$), MDA-MB-468 ($p53^{R273H}$), and Hs578T ($p53^{V157F}$) transfected with NS siRNA or p53 siRNA were analyzed by western blotting. NS siRNA or p53 siRNA transfected Hs578T **(B)**, MDA-MB-231 **(C),** and MDA-MB-468 **(D)** cells were treated with DMSO or DPEITC for 72 h. Percent cell proliferation was determined by the WST-1 assay. Experiment were performed in triplicate. Error bars represents SD.

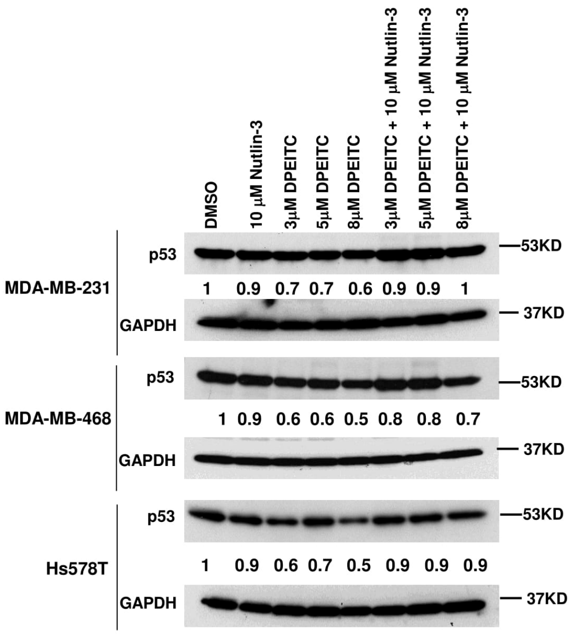

Figure S4. DPEITC sensitizes p53 mutants to proteasomal degradation. MDA-MB-231, MDA-MB-468, and Hs578T cells were treated DPEITC (3, 5, or 8 μM), Nutlin-3 (10 μM), or both for 4 h. Cells were harvested and lysates were prepared. Lysate fractions were resolved by SDS-PAGE and probed with p53 DO-1 antibody. Experiment were performed in triplicate.

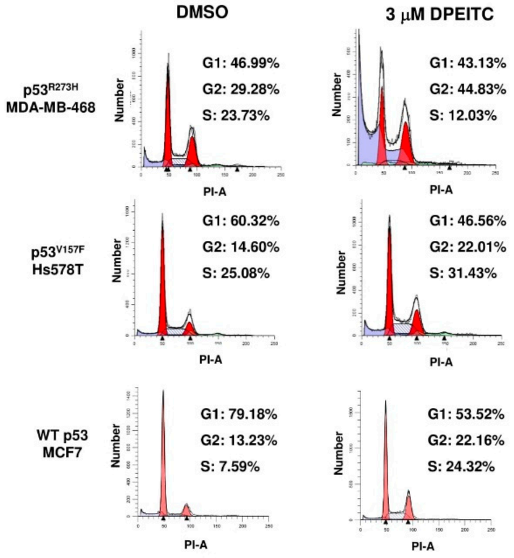

Figure S5. DPEITC induces a cell cycle delay in breast cancer cell lines. MCF7 (WT p53), MDA-MB-468 ($p53^{R273H}$), and Hs578T ($p53^{V157F}$) cells were treated with DMSO or 3 μM DPEITC for 72 h and analyzed by flow cytometry. Representative cell cycle analysis images are shown.

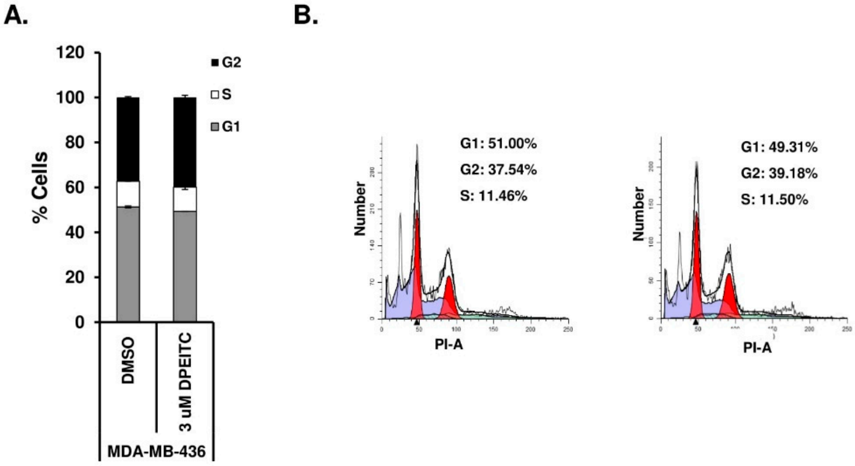

Figure S6. Effects of DPEITC on cell cycle progression in p53 null MDA-MB-436 TNBC cell line. **(A)** p53 null MDA-MB-436 cells were treated with DMSO or 3 μM DPEITC for 72 h and analyzed by flow cytometry. Experiment were performed in triplicate. Error bars represents SD. **(B)** Representative cell cycle images are shown.

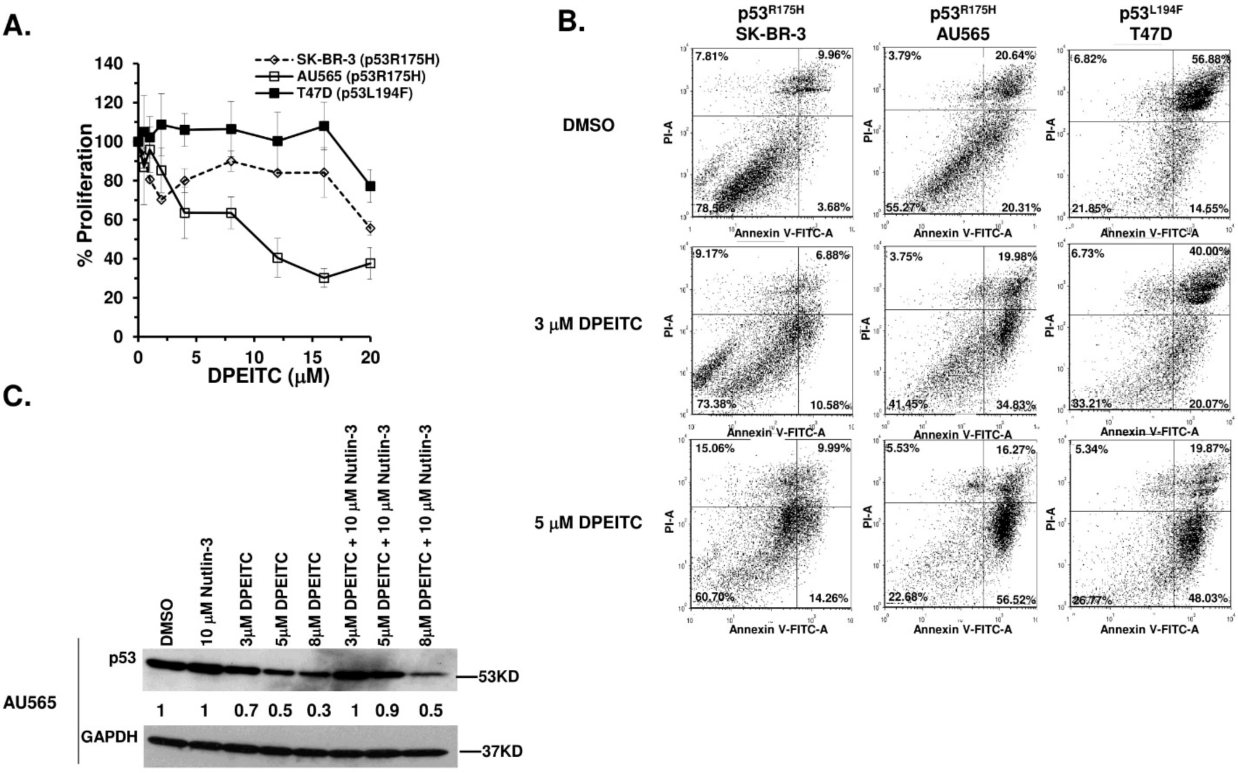

Figure S7. Effects of DPEITC on the proliferation, apoptosis, and mutant p53 expression levels in HER2+ and Luminal A breast cancer cell lines. (**A**) HER2+ SK-BR-3 ($p53^{R175H}$) and AU565 ($p53^{R175H}$), and Luminal A T47D ($p53^{L194F}$) cell lines were treated with DMSO or DPEITC for 24 h. Percent cell proliferation was determined by the WST-1 assay. (**B**) HER2+ SK-BR-3 ($p53^{R175H}$) and AU565 ($p53^{R175H}$), and Luminal A T47D ($p53^{L194F}$) cell lines were treated with DMSO or DPEITC for 72 h and analyzed for apoptosis. (**C**) AU565 ($p53^{R175H}$) were treated DPEITC (3, 5, or 8 μM), Nutlin-3 (10 μM), or both for 4 h. Cells were harvested and lysates were prepared. Lysate fractions were resolved by SDS-PAGE and probed with p53 DO-1 antibody. Experiment were performed in triplicate. Error bars represents SD.

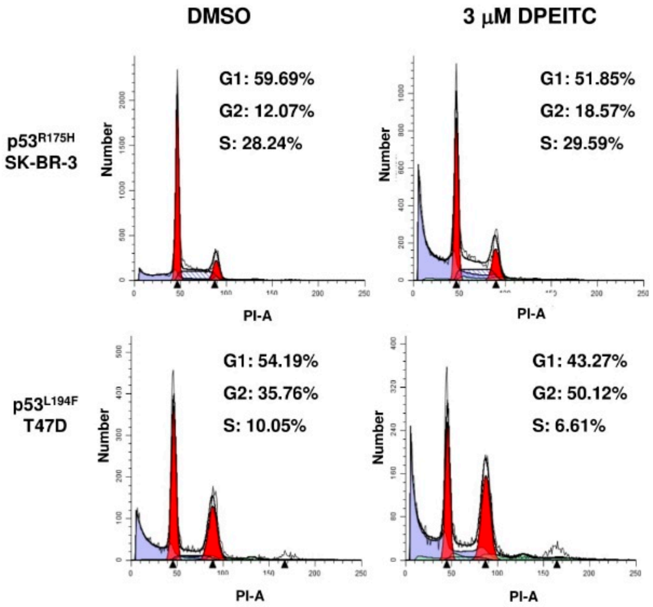

Figure S8. DPEITC induces a cell cycle delay in HER2+ and Luminal A breast cancer cell lines. SK-BR-3 ($p53^{R175H}$) and Luminal A T47D ($p53^{L194F}$) cells were treated with DMSO or 3 μM DPEITC for 72 h and analyzed by flow cytometry. Representative cell cycle analysis images are shown.

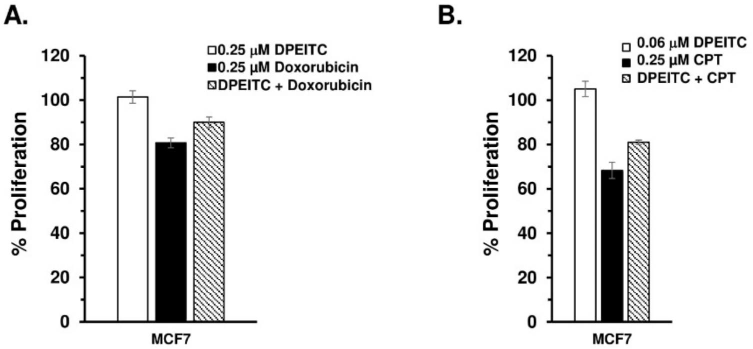

Figure S9. Effects of DPEITC and topoisomerase inhibitor co-treatment on WT p53 MCF7 cell proliferation. (**A**) WT p53 MCF7 cells were treated with DPEITC, doxorubicin, or both for 24 h. (**B**) WT p53 MCF7 cells were treated with DPEITC, CPT, or both for 24 h. Percent cell proliferation was determined by the WST-1 assay. Experiment were performed in triplicate. Error bars represents SD.

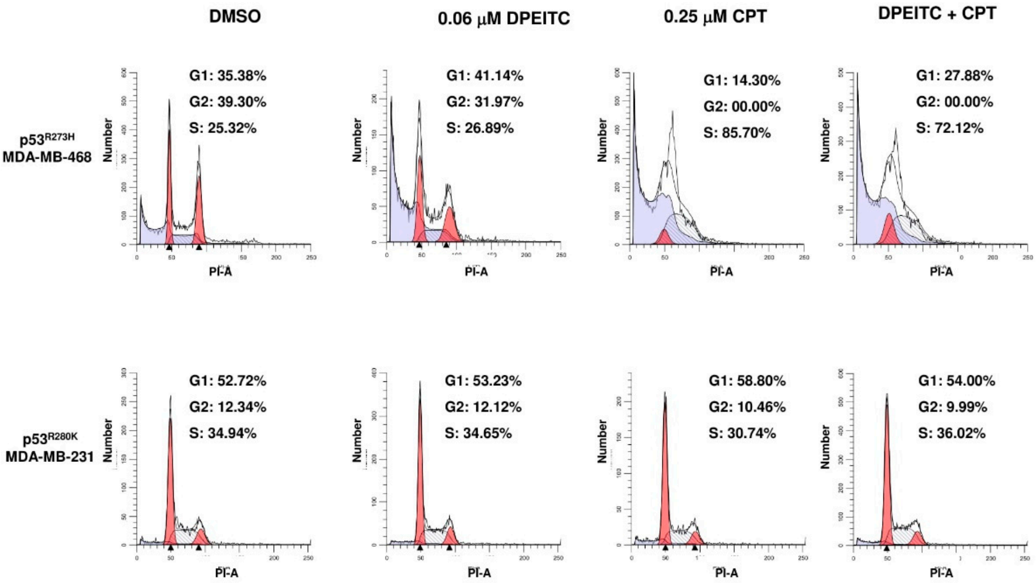

Figure S10. Effects of DPEITC and CPT co-treatment on cell cycle progression in p53 mutant TNBC cell lines. MDA-MB-468 ($p53^{R273H}$) and MDA-MB-231 ($p53^{R280K}$) cells were treated with DMSO, 0.06 μM DPEITC, 0.25 μM CPT, or both for 24 h and analyzed by flow cytometry. Representative cell cycle analysis images are shown.
